# The prevalence of polycystic ovary syndrome in adolescents: A systematic review and meta-analysis

**DOI:** 10.18502/ijrm.v17i8.4818

**Published:** 2019-09-03

**Authors:** Marzieh Saei Ghare Naz, Fahimeh Ramezani Tehrani, Hamid Alavi Majd, Fazlollah Ahmadi, Giti Ozgoli, Farzaneh Rashidi Fakari, Vida Ghasemi

**Affiliations:** ^1^ Student Research Committee, Department of Midwifery and Reproductive Health, School of Nursing and Midwifery, Shahid Beheshti University of Medical Sciences Tehran Iran.; ^2^ Reproductive Endocrinology Research Center, Research Institute for Endocrine Sciences, Shahid Beheshti University of Medical Sciences Tehran Iran.; ^3^ Department of Biostatistics, School of Allied Medical Sciences, Shahid Beheshti University of Medical Sciences Tehran Iran.; ^4^ Department of Nursing, Faculty of Medical Sciences, Tarbiat Modares University Tehran Iran.; ^5^ Department of Midwifery and Reproductive Health, School of Nursing and Midwifery, Shahid Beheshti University of Medical Sciences Tehran Iran.; ^6^ Student Research Committee, Midwifery and Reproductive Health Research Center, Shahid Beheshti University of Medical Sciences Tehran Iran.

**Keywords:** Polycystic ovary syndrome, Prevalence, Meta-analysis, Adolescence

## Abstract

**Background:**

Polycystic ovarian syndrome is an endocrine disorder with many complications. This syndrome is a growing concern among adolescents around the world, with varying reports of its prevalence in different parts of the world.

**Objective:**

This study aimed to determine the prevalence of polycystic ovary syndrome in adolescents by a systematic review and meta-analysis.

**Materials and Methods:**

In this study, a search for published articles with an English language limitation and without a time limit was done in different databases (Scopus, PubMed, and Web of Science, Emabse and Cochrane) in January 2019. The 12 studies that met the criteria for entering a qualitative assessment scale of 5 and higher were subjected to systematic review and meta-analysis. Egger and Begg's tests were used to check the publication bias. Data were analyzed with STATA software, version 11.1.

**Results:**

Twelve studies were included for meta-analysis. The total number of participants in the study was 149,477. The average quality score of all studies was 8.67 (range: 5–10). The prevalence of polycystic ovarian syndrome in adolescents based on the Rotterdam criteria was 11.04% (95% CI: 6.84–16.09%), based on the National Institute of Health criteria, it was 3.39% (95% CI: 0.28–9.54%), and based on Androgen Excess and Polycystic Ovary Syndrome Society, it was 8.03% (95% CI: 6.24–10.01%)

**Conclusion:**

The result of this study showed that there is a variation in the prevalence of PCOS in adolescents based on different criteria; we suggest more community-based studies among adolescences in different parts of the world.

## 1 Introduction

Polycystic ovary syndrome (PCOS) is a common endocrine disorder in reproductive age women (1, 2). According to the systematic review and meta-analysis the prevalence of PCOS in women of reproductive age estimated from 5% to 18% (3). PCOS is diagnosed by hyperandrogenism, ovarian disorder, and polycystic ovaries, although there are significant variations between individuals (4). This multi-factorial syndrome initially appears in puberty (5), and individuals with this disease may be exposed to the risk of several diseases, including obesity, metabolic syndrome, insulin resistance, type-II diabetes, infertility, cancer, cardiovascular disease, and mental disorders (6, 7), affecting several dimensions of the quality of life (8). The evidence suggest that this syndrome is a disorder presenting in adolescents due to genetic ovarian malfunction that leads to the excessive secretion of androgens, and there is evidence for a genetic basis of PCOS during the life of the fetus and physiological hypothalamus-pituitary activation of the ovaries in the neonatal period and at the beginning of puberty (9). Congenital venous disorders, higher-than-average or low birth weight during pregnancy and childbirth, premature adrenarche, obesity with acanthosis nigricans, metabolic syndrome, and pseudo-Cushing’s syndrome or pseudo acromegaly in early childhood are known as independent risk factors before menstruation for developing PCOS (10).

The criteria for the diagnosis of this syndrome are defined in accordance with various organizations. The National Institute of Health (NIH) has designed the NIH criteria, the American Society of Reproductive Medicine has designed the Rotterdam criteria, and the Androgen Excess and Polycystic Ovary Syndrome Society (AE-PCOS) has designed the AES criteria (11–13). This heterogeneous disorder leads to the excess production of androgens especially from the ovaries, also it is associated with lack of ovulation, hirsutism, and insulin resistance (14) that is a relatively common disorder among teenage girls. The common clinical features such as hirsutism and menstrual irregularities usually do not appear until mid- to late adolescence (15). In adolescent girls, natural characteristics of maturity overlap with the signs and symptoms of PCOS (16, 17). Three different sets of diagnostic criteria use to define the disease in mature women, but there is a debate on the use of these criteria in adolescents (18), and it seems the existence of all three groups of symptoms is essential for the diagnosis of this syndrome in this younger population. Hence, etiopathogenic and diagnostic criteria challenges for PCOS in teens continues (19).

Reports relating to the prevalence of PCOS in adolescents are rare (20, 21). In a study in India, the prevalence rate of this syndrome in 15–19-year-old adolescents based on the Rotterdam criteria was 22.6% and based on the AE-PCOS criteria it was 9.8% (22). In another study on adolescents aged 17–19 in Thailand, the prevalence rate of this syndrome was 5.29% (23). Studies in Iran have shown the prevalence rate to be 8.3% to 11.4% (24, 25).

Despite scattered studies in various parts of the world, this meta-analysis and systematic review evaluates the prevalence of PCOS in adolescent girls worldwide.

## 2 Materials and Methods

### 2.1 Search strategy 

The search in this meta-analysis and systematic review was performed by two researchers. Published articles with an English language limitation and without a time limit were searched in the databases such as Scopus, PubMed, Web of Science, Emabse, and Cochrane in January 2019. The search strategy was as follows:

(Prevalence OR Epidemiology OR Cross-Sectional OR Cross-Sectional Analyses OR Cross Sectional Analysis AND Adolescence OR Adolescents OR Female Adolescent OR Teenager OR Youth OR Teens OR Student AND Polycystic Ovary Syndrome OR PCOS OR Ovary Syndrome, Polycystic OR Ovarian Syndrome, Polycystic OR Stein-Leventhal Syndrome Sclerocystic Ovary OR Ovary, Sclerocystic OR Sclerocystic Ovaries).

After searching in the aforementioned databases, 1,888 articles in the first search were imported into Endnote and after excluding duplications (n░=░996) and articles with irrelevant topics (n░=░869), finally, 12 articles were included in the meta-analysis.

### 2.2 Risk of bias

The quality assessment of the articles was conducted with a valid tool used for the data related to prevalence. This quality assessment tool consists of 10 items and each item has three options “Yes, No, or Non-transparent.” The range of scores is 0–10 and where the option “Yes” is applied, the score is 1 and for other cases, zero is applied. Studies with a score of 5 and higher were included in the study. In this scale, sample representative, sample size, study subjects, data analysis measurement criteria, and the overall methodological methodological of the study were examined (26). Table I shows the results of the quality assessment and its scores.

### 2.3 Selection of studies and data extraction

**Figure 1:**
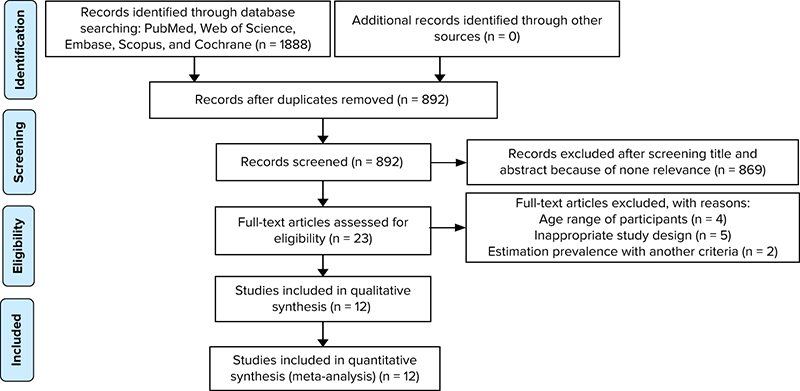
Flow diagram of included studies.

Next, we reviewed the titles and abstracts of the articles, and after the removal of irrelevant articles, the full texts of the relevant articles were extracted and examined. The diagram of the selected studies is shown in Figure 1.

After the quality assessment of articles, data extraction was conducted. The extraction of the data related to prevalence was conducted by two researchers on the basis of the standard NIH, AE-PCOS Society criteria and Rotterdam criteria. The data extraction form included the author of the study, year of the study, place of the study, type of the study, the population of the study and its features, prevalence of PCOS based on different criteria, and the prevalence of acne, hirsutism, and menstrual disorders.

### 2.4 Eligibility criteria for inclusion of the study

The observational studies that were included in the meta-analysis had the following characteristics: (1) they were conducted on 10–20-year-old females, (2) PCOS diagnosis was based on one of the standard criteria (NIH, Rotterdam, and AE-PCOS), and (3) study samples had no known disorders (such as Cushing’s, thyroid disorder, and so forth).

### 2.5 Outcomes

In this study, the primary and main outcome was the prevalence of PCOS according to the standard criteria, and the secondary outcome was the prevalence of hirsutism, acne, and oligomenorrhea.

### 2.6 Statistical analysis

The effect size in this study was the prevalence of PCOS in adolescence, and its variance (with 95% confidence interval) was calculated using the binomial distribution. The I^2^ index (I^2^ statistic) was calculated for heterogeneity. I^2^ >50% indicates significant heterogeneity. In cases where the studies were heterogeneous, the random effects model was used to estimate the pooled prevalence. Egger and Begg's tests were used to check publication bias (35). The heterogeneity between the results of the studies was analyzed using the Chi squared (χ2) test at a significant level of 5% (p░<░0.05). We used Metaprop command in STATA for the stability of variance (36). STATA software (version 11.2) was used to analyze the data.

## 3 Results

This study aimed to determine the prevalence of PCOS in adolescent girls. The PRISMA checklist was used for writing of this study (37). Twelve studies were included in this meta-analysis study. The average quality score of all studies was 8.67 (range: 5–10).

The total number of participants in the studies was 149,477. The mean age of the participants was 16.99 years (95% CI: 16.46–17.52), average body mass index was 21.09 (95% CI: 20. 3–21.88) kg/m^2^. Five studies were related to Iran, four to India, one to Thailand, one to the United States, and one to Australia.

### 3.1 Primary outcomes

**Figure 2:**
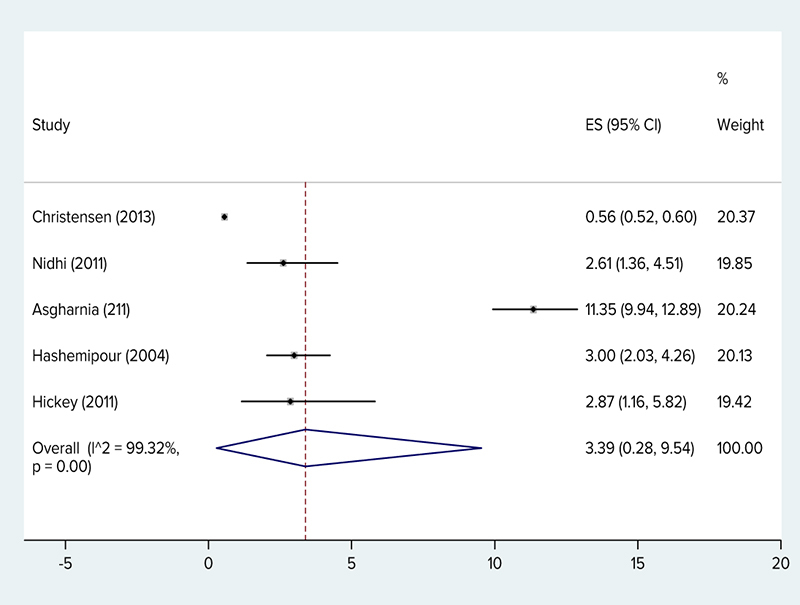
The prevalence of polycystic ovary syndrome (NIH criteria) by researcher, year, prevalence and 95% confidence interval in the world.

**Figure 3:**
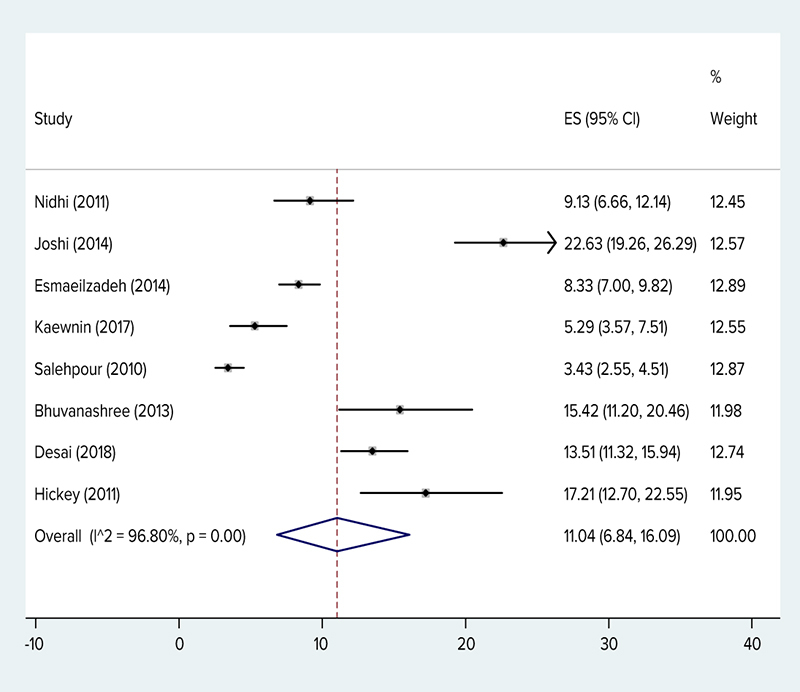
The prevalence of polycystic ovary syndrome (Rotterdam criteria) by researcher, year, prevalence and 95% confidence interval in the world.

The results of this study showed that the prevalence of PCOS in adolescents was 11.04% (95% CI: 6.84–16.09%) based on the Rotterdam criteria and I^2^ was 96.80%. The NIH criteria was 3.39% (95% CI: 0.28–9.45%) and I^2^ was 99.32%, based on AES was 8.03% (95% CI: 6.24–10.01%). Figures 2 and 3 show overall prevalence.

Additionally in three studies (23, 28, 31), prevalence based on the Rotterdam criteria by phenotype clinical hyperandrogenism and oligomenorhea (OA, HA) was 0.47% (95% CI: 0.1–1.05%), clinical hyperandrogenism phenotype and Polycystic ovary (HA, PCO) was 1% (95% CI: 0.38–1.89%), oligomenorhea and Polycystic ovary (OA, PCO) was 2.6% (95% CI: 0.67–5.68%), and clinical hyperandrogenism phenotype and Polycystic ovary and oligomenorhea (HA, OA, PCO) was 1.56% (95% CI: 0.44–3.29%).

**Figure 4:**
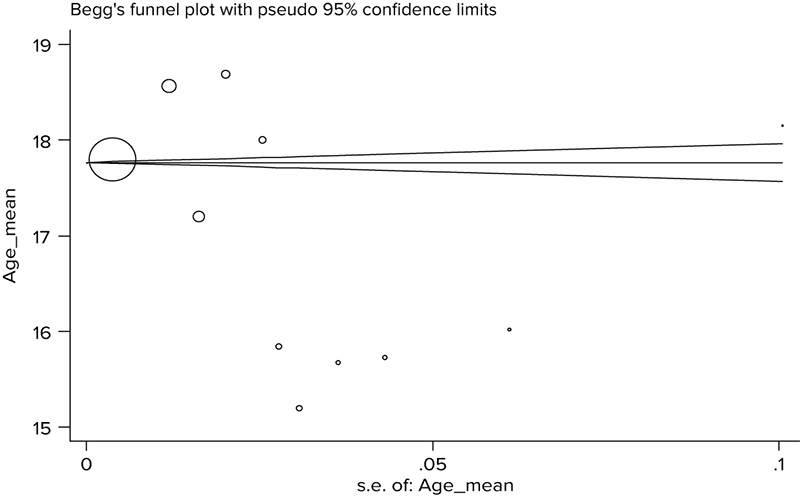
Funnel plot for checking publication bias.

Table I shows the characteristics of the included studies. Based on the results of the Begg's test (p░=░0.244) and Egger's test (p░=░0.155), publication biases did not exist in the studies (Figure 4).

**Table I:** The characteristics of the included studies

Author (year) (ref no.)	Location	Type of study	Participants	Prevalence for PCOS			Quality assessment
				NIH criteria	Rotterdam’s criteria	AES criteria	
Joshi (2014) (22)	India	Community-based cross-sectional	N░=░570 15–19 years	-	22.60%	9.80%	9
Kaewnin (2017) (23)	Thailand	Cross-sectional study	N░=░548 17–19 years	-	5.29% (HA, OA) were presented in 1 cases, (HA, PCO) in 8 cases, (OA, PCO) in 8 cases, and (HA, OA, and PCO) in 12 cases	-	10
Asgharnia (2011) (24)	Iran	Cross-sectional study	N░=░1850 17–18 years	11.34%	-	-	5
Esmaeilzadeh (2014) (25)	Iran	Cross-sectional study	N░=░1549 16–20 years		8.3% (95% CI; 4.0, 12.0)		8
Desai (2018) (27)	India	cross-sectional community-based	N = 881 13–18 years	-	13.54%	-	9
Akbarzadeh (2015) (28)	Iran	Cross-sectional study	N = 3190 14–18 years	-	(HA, OA) were presented in (19.9%), (HA, PCO) in (30.8%), (OA, PCO) (29.5%), and (HA, OA, and PCO) in 21 cases (14.5%)	-	9
Christensen (2013) (29)	Southern California	Cross-sectional study	N░=░137,502 15 -19 years	0.56% (0.52%-0.60%)	-	-	10
Bhuvanashree (2013) (30)	India	Community-based cross-sectional	N = 253 10–19 years	-	15.4% (95% CI, 10.97–19.83)	-	10
Nidhi (2011) (31)	India	-	N = 460 15–18 years	2.61%	9.13% (HA, OA) were presented in (0.22%), (HA, PCO) in (0.22%), (OA, PCO) (6.30%), and (HA, OA, and PCO) in (2.39%)	-	9
Hickey (2011) (32)	Australia	Prospective cohort study	N░=░244 girls 14–16 years	3.10%	18.50%	5%	10
Salehpour (2010) (33)	Iran	Cross-sectional study	N░=░1430 15–18 years	-	3.42%	-	8
Hashemipour (2004) (34)	Iran	Cross-sectional study	N = 1000 14–18 years	3%	-	-	7

### 3.2 Secondary outcomes

The prevalence hirsutism in girls with PCOS in five studies was 18%, the prevalence of acne in two studies was 35%, and oligomenorrhea in two studies was 79%.

## 4 Discussion

A total of 149,477 girls participated in this meta-analysis and systematic review. According to the data, PCOS in adolescents was 11.04% (95% CI: 6.84–16.09%) based on the Rotterdam criteria. The NIH criteria was 3.39% (95% CI: 0.28–9.45%), and based on AES was 8.03% (95% CI: 6.24–10.01%). In a study on 126 Qatari females (age range: 18–30 years), the prevalence of this syndrome based on the NIH criteria was 33.18% (38). In a study done on the Iranian population (age range: 17–34 years old), the prevalence based on the NIH criteria was 7%, based on the Rotterdam criteria it was 15.2%, and based on the AES criteria it was 7.92% (39). In a study by Ramezani Tehrani and colleagues on a sample of Iranian women with the average age of 34.4 +/- 7.6 years, the prevalence rate based on the NIH criteria was 7.1% (95% CI: 5.4–8.8%) (40). In a study by Ybarra and colleagues done on 49 obese Brazilian adolescents, the prevalence rate of PCOS based on the Rotterdam criteria was 26.4%, based on the AES criteria it was 22.4%, and based on the NIH criteria it was 20.4% (41). In a study on 16- to 29-year-old Australian youth, the prevalence rate based on the NIH criteria was 12% (41). In the meta-analysis study by Ding and colleagues, the prevalence of PCOS in women of different races showed that the prevalence rate in Chinese women was 5.6% (based on the Rotterdam criteria), which was the lowest prevalence rate, and in women of Middle Eastern countries it was 16% based on the Rotterdam criteria (3). A meta-analytic study in Iran showed the prevalence of PCOS in Iranian women was 19.5% based on the Rotterdam criteria (42). In a systematic review and meta-analysis by Bozdag and colleagues on women of reproductive age excluding adolescents, the prevalence rate based on the Rotterdam criteria was 10% (n░=░15 trials) and based on the NIH criteria the prevalence rate was 6% (n░=░18 trials) (43). It seems the prevalence of this syndrome in adolescents is almost the same as adults. Also, as in the study by Bozdag and colleagues, the prevalence of this syndrome based on the Rotterdam criteria was double that of the NIH (43). A study by Rashidi and colleagues reported that the prevalence of PCOS was 2.9 times more according to the Rotterdam criteria when compared with the NIH criteria (44). Although the number of adolescent studies is few, the evidence shows that the difference in estimates of prevalence is in part due to the diagnostic criteria (45). In adolescents, the heterogeneity of clinical symptoms of PCOS and the lack of uniformity in the definition of the symptoms hinder diagnosis. Moreover, the global use of PCOS diagnostic criteria is varied (46) and in general, there is no consensus on how to define PCOS for teenagers (47). The PCOS signs are different based on different characteristics such as the age, race, and weight and add to the challenges of accurate diagnosis. In adolescents natural maturity characteristics usually overlap with signs and symptoms of PCOS, this issue lead to particular diagnostic problems (16, 48), and based on the evidence, the debate on the etiopathogenesis, diagnostic criteria, and suggestions for PCOS in adolescents continues (19). Moreover, the diet and lifestyle of individuals in different geographical regions affect the prevalence rate of this syndrome in various regions (49). In this study, the prevalence hirsutism in PCOS girls in five studies was reported as 18%, the prevalence of acne in two studies was 35%, and oligomenorrhea in two studies was reported as 79%. In a study by Ramezani Tehrani and colleagues, the estimated prevalence of idiopathic hirsutism and menstrual disorder was 13% and 1.5%, respectively (50). Nonetheless, in the meta-analytic study of women in reproductive age (excluding adolescents), the prevalence of hirsutism was 13% (8–20%, n░=░14 trials), the prevalence of acne was 16% (8–26%, n░=░12 trials), and the prevalence of alopecia was 2% (0–5%, n░=░5 trials) (43). In fact, these results indicate the prevalence of secondary consequences like acne and hirsutism was more than that of the adults reported in the study by Bozdag and colleagues (43).

### 4.1 Limitation

A limitation of the study was that the number of studies was based on a small population. In addition, some diagnostic criteria such as hirsutism fluctuate among different races. Also, diagnostic challenges during adolescence, as well as the fact that most studies were restricted to Iran, were the other biases of this study. Being limited to the English language in searching articles caused restrictions as well.

## 5 Conclusion

The result of this study showed that there is a variation in the prevalence of PCOS among adolescents based on different criteria; we suggest more community studies among adolescences in different parts of the world.

## Conflict of Interest

The authors declare no conflict of interest.
